# Lightness dependence of achromatic loci in color-appearance coordinates

**DOI:** 10.3389/fpsyg.2015.00067

**Published:** 2015-02-10

**Authors:** Ichiro Kuriki

**Affiliations:** Research Institute of Electrical Communication, Tohoku UniversitySendai, Japan

**Keywords:** color vision, color constancy, achromatic point, lightness dependence, corresponding colors

## Abstract

Shifts in the appearance of color under different illuminant chromaticity are known to be incomplete, and fit nicely with a simple linear transformation of cone responses that aligns the achromatic points under two illuminants. Most chromaticity-transfer functions with von-Kries-like transformations use only one set of values to fit the color shifts from one illuminant to another. However, an achromatic point shifts its chromaticity depending on the lightness of the test stimulus. This lightness dependence of the achromatic-point locus is qualitatively similar to a phenomenon known as the Helson-Judd effect. The present study suggests that the lightness dependency of achromatic points appears to be a general trend, which is supported by the results from deriving the optimal von-Kries coefficients for different lightness levels that best fit the color shifts under a different illuminant chromaticity. Further, we report that such a lightness dependence of the achromatic-point loci can be represented simply as a straight line in coordinates defined using color-appearance models such as CIECAM when normalized for daylight.

## Introduction

Several studies on color-constancy have reported that shifts in color appearance under changes in illuminant chromaticity are often incomplete. Shifts in an achromatic point have been used as a measure to investigate changes in the sensitivity of color vision, which may result in shifts in color appearance. Some previous studies have reported that shifts in color appearance can be fitted using a simple linear scaling of the cone excitations, such that the chromaticity of light perceived as achromatic (i.e., achromatic points) under two illuminants will be aligned in the cone-response space (Speigle and Brainard, 1999; Kuriki et al., 2000). Linear transformations of cone excitations using a 3 × 3 diagonal matrix are termed a *von-Kries type* or *von-Kries transformation*, after the discovery by Johannes von Kries (1970) The diagonal entries of a 3 × 3 matrix are often called *von-Kries coefficients*, which only vary the gain of three photoreceptors, i.e., the L, M, and S cones. Our previous study reported that shifts in the appearance of color chips under a different illuminant chromaticity are nicely fitted using a scaling of the cone responses (Kuriki et al., 2000), with the von-Kries coefficients derived by shifts in the achromatic point.

On the other hand, several studies have reported that the locus of an achromatic point depends on the lightness level of the test stimulus. The so-called Helson-Judd effect is a phenomenon in which spectrally non-selective (i.e., achromatic) surfaces under monochromatic light appear different in hue for lighter and darker samples (Helson, 1938; Judd, 1940; Helson and Michels, 1948). Helson (1938) first reported that lighter samples appear to have the same hue as that predicted by the wavelength of a monochromatic illuminant, whereas darker samples appear to have a hue opposite to what is predicted. Because monochromatic light has only a single wavelength component, the only factor that changes in a space illuminated with monochromatic light is the energy of the reflected light. Therefore, if the color appearance is a consequence of linear transformations of the cone responses, the apparent hue of the object, illuminated under a monochromatic light, should not vary with the intensity of the reflected light. Therefore, the Helson-Judd effect implies that *an achromatic point should be lightness dependent*; in other words, an achromatic point should be less saturated than the illuminant chromaticity for higher-reflectance samples, and more saturated than the illuminant chromaticity for lower-reflectance samples. Helson and Michels (1948) reported a phenomenon of systematic dependence of the locus of an achromatic point on the lightness of the test sample, and some recent studies have also reported the dependence of an achromatic point on the intensity of the test stimulus in relation to the surrounding surfaces (e.g., Bäuml, 2001; Kuriki, 2006).

If the alignment of the achromatic points under a different illuminant chromaticity predicts a shift in the color appearance of color chips with the same lightness, different von-Kries coefficients should be derived for the different lightness levels. However, the von-Kries coefficients applied in previous studies were derived using only a single pair of achromatic points under two different illuminant conditions (Speigle and Brainard, 1999; Kuriki et al., 2000), and did not take the lightness dependence into account. Thus, a simple question arises: *is there a contradiction between our two studies?*

The first half of this paper demonstrates that the loci of achromatic points are lightness dependent. As a part of this demonstration, we use data from two studies (Kuriki et al., 2000; Kuriki, 2006) to show that these two reports are not subject to discrepancies. We first evaluate the lightness dependency by deriving the optimal von-Kries coefficients for each lightness level in the data for the asymmetric color-matching experiment (Kuriki et al., 2000). We then compare these results with our later study reporting the lightness dependence of an achromatic point (Kuriki, 2006). In the second half of this paper, we provide a supplementary exploration by plotting the achromatic-point loci used in the previous study (Kuriki, 2006) at various coordinates defined using color-appearance models. We report that the achromatic loci, measured under various illuminant colors, appear nearly parallel with each other along the lightness axis when the color space is normalized to a pure-white (i.e., spectrally non-selective) surface with 100% reflectance in daylight.

It must be carefully stated that the *achromatic points* used in this and previous studies (Speigle and Brainard, 1999; Kuriki et al., 2000; Kuriki, 2006) represent a chromaticity of light that gives a colorless appearance to the subjects. On the other hand, a color of light that yields zero chromatic components (i.e., lightness axis) in the color-appearance models implies that the color *matches* the apparent color of the achromatic chips under the tested illuminant; in other words, they do not always represent the points of an achromatic (i.e., colorless) *subjective* appearance because the color appearance models were developed to predict shifts in the benchmark data of the “corresponding colors,” and were clearly not optimized for the prediction of achromatic loci (Fairchild, 2013).

In addition, the definitions of the terms *intensity*, *luminance*, and *lightness* used in the present study should be clarified. Herein, “intensity” indicates the strength of light in a general sense, and can include the notions of both luminance and lightness. The word “luminance” is a psychophysical scale indicating the intensity of light, and can be derived physically by integrating the spectrum of light incident to the eye, multiplied by the luminosity efficiency function, *V*(λ), across the visible wavelength spectrum using an appropriate scaling factor. The luminance is a physically measurable value and is proportional to the energy of light. The terms “lightness” refers to the perceived strength of light from an object surface with respect to the perceived strength of light of another area that shows a high-reflectance of *white* under the same illumination. The lightness is proportional to the luminance (or energy) of light to the 1/3rd power, which is referred to as the *1/3 power-law*, and is adopted as the intensity axis in the CIE LAB space (1976) defined by the International Commission on Illumination (CIE). For more formal definitions of these words, see CIE's list of terms (http://eilv.cie.co.at/).

Models for a color-appearance transformation have been rigorously tested by technical committees at CIE. CIE has proposed several numerical models that satisfactorily match the shifts in the appearance of color chips, observed under standard illuminants, mainly between standard illuminants A and D_65_ (Fairchild, 2013). However, the present study does not debate quantifying the color appearance, such as the lightness, hue, and chromatic saturation, of color chips. Rather, the present study focuses on introducing some suggestions for a particularly general view of how the *neutral point of color appearance* changes with the illuminant colors. To do so, we take a much wider range of illuminant colors into account, including red-green directions (Kuriki, 2006). As a part of such a general view on the sensitivity changes in color vision, the achromatic loci used in our previous study were tested in spaces defined by color-appearance models such as CIELAB (1976), CIECAM97s (revised version by Fairchild, 2001), CIECAM02 (2002), Nayatani's model (Nayatani et al., 1990), Hunt's model (Hunt, 1994), and RLAB (Fairchild, 2013), the results of which are described in the latter half of this study.

## Confirmation of the lightness dependence for achromatic loci

### Rationale

**Figure 1:**
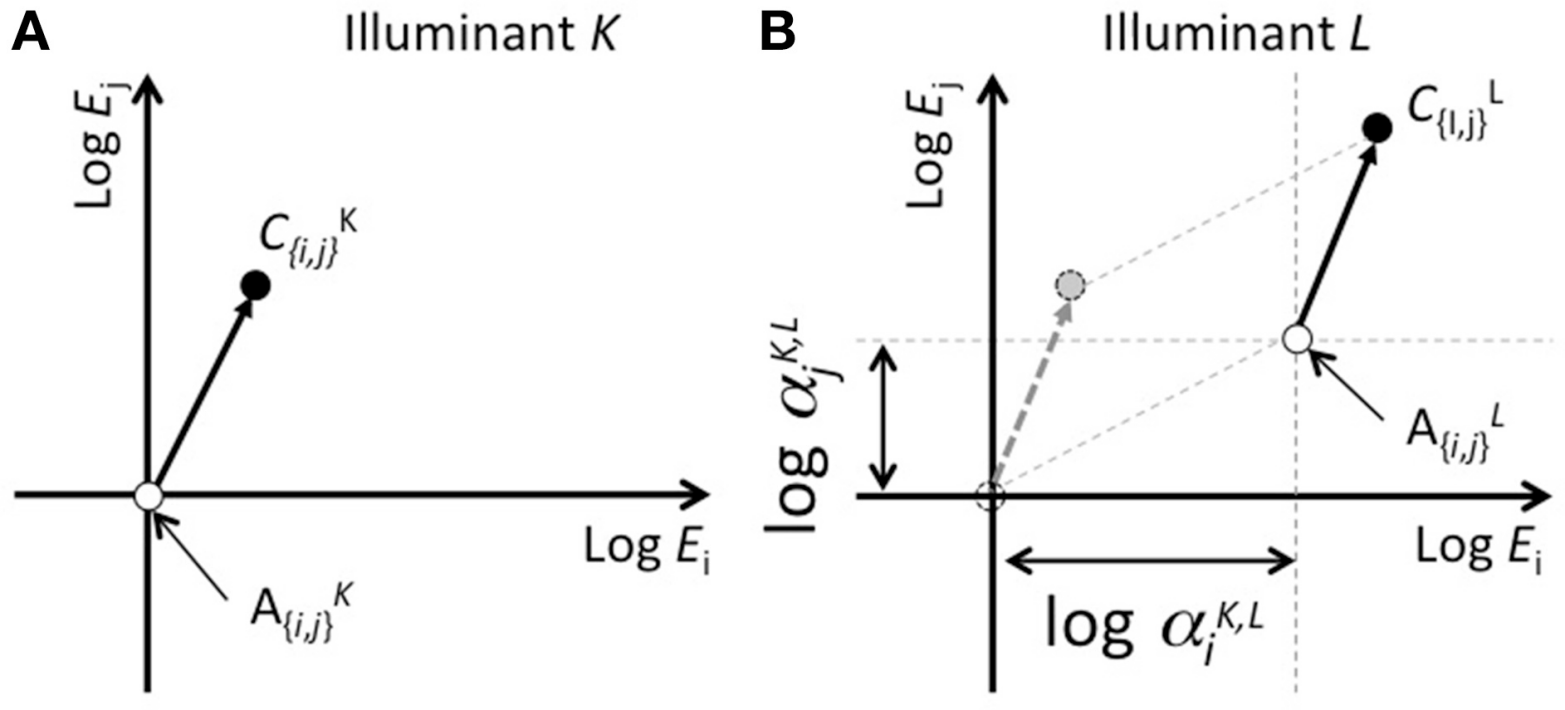
**Schematic of the concept of color-shift predictions from scaling cone responses in accordance with the achromatic point. (A,B)** represent color appearance in cone response space under illuminants *K* and *L*, respectively. The vertical and horizontal axes represent logarithms of the responses for two cone classes. The scaling of the cone response is represented by a parallel shift in this plane. The difference between the achromatic points under two illuminant conditions (*alpha*^*K,L*^_{*i, j*}_, where *i* and *j* represent cone classes, and *K* and *L* represent illuminant differences) represents logarithms for the von-Kries coefficients.

If the alignment of achromatic-point shifts in the cone excitations (Speigle and Brainard, 1999; Kuriki et al., 2000) successfully predicts shifts in the color appearance under different illuminants, the von-Kries coefficients that minimize the differences between the appearance and the predicted colors will represent shifts in the achromatic points. A search for the optimal von-Kries coefficient was conducted using our previous data (Kuriki et al., 2000) at each of three lightness levels (Munsell *Values* of 3, 5, or 7). The prediction of the color appearance for the corresponding color chips under a particular illuminant was derived from the color appearance, matched under a standard white (D_65_) illuminant, by applying the von-Kries coefficients. Figure 1 illustrates the concept of a color-shift model under this configuration.

Figure 1 shows an example of an achromatic point and the appearance of a color chip *C* by taking the logarithm of the cone response as the axes. In this space, a von-Kries-like transformation can be represented as a parallel shift, and the degree of shift along each axis corresponds to the coefficients for each cone response. For simplicity, only two of the three dimensions are shown. The origin of the axes are normalized to the achromatic point under illuminant *K*. Figures 1A,B represent the color appearance under illuminants *K* and *L*, respectively. The open and filled symbols indicate the achromatic point and appearance of the color chip *C*, respectively, under each illuminant. In Panel Figure 1B, the dotted lines parallel to the axes indicate a shift in the achromatic point from illuminants *K* to *L*. Given that *W*^{*K,L*}^_{*i,j*}_ represents the coordinates (either *i* or *j*) for the achromatic point under illuminants *K* or *L*, the scaling coefficient that aligns the achromatic points is defined as follows:

(1) $$\alpha_{\left\{i,j\right\}}^{K,L}=W_{\left\{i,j\right\}}^{L}/W_{\left\{i,j\right\}}^{K}$$

The application of this coefficient to the coordinates of the color chip can be represented as follows:

(2) $${\hat{C}}_{i,j}^{L}=\alpha_{\left\{i,j\right\}}^{K,L}C_{\left\{i,j\right\}}^{K},$$

where $\hat{C}$^*L*^_{*i,j*}_ represents the calculated coordinates for cone class *i* or *j* under illuminant L after scaling the cone responses.

In our previous study, the subjects matched the color appearance under different chromatic illuminants by adjusting the chromaticity of a color stimulus on a CRT surface, shown through a hole in a mid-level gray wall under a standard illuminant, D_65_ (see Kuriki et al., 2000 for details). The matched colors under D_65_ represent the appearance of color chips under the chromatic illuminants tested.

We calculated the *optimal* von-Kries coefficients for each of the three lightness levels such that the color differences between the matched and calculated colors were minimized in CIE delta-E^*^. To confirm the consistency with our previous study, the coefficients obtained in the results were plotted in the same way as in **Figures 4**, **5** of our previous study on the loci of the achromatic points (Kuriki, 2006).

### Procedure

The following procedure was adopted. First, an illuminant was selected for testing using a set of 20 color chips from our previous study (Kuriki et al., 2000). We chose the cone excitations that match the chromaticity for each color chip under the tested illuminant, and the cone excitations for the control match (D_65_ to D_65_) of the color chip. The following simple von-Kries-like transformation was applied:

(3) $$\left[\begin{array}{c} {\hat{L}}_{i}^{j}\\ {\hat{M}}_{i}^{j}\\ {\hat{S}}_{i}^{j} \end{array}\right]=\left[\begin{array}{ccc} \alpha_{k}^{D_{65,j}} & 0 & 0\\ 0 & \beta_{k}^{D_{65,j}} & 0\\ 0 & 0 & \gamma_{k}^{D_{65,j}} \end{array}\right]=\left[\begin{array}{c} L_{i}^{D_{65}}\\ M_{i}^{D_{65}}\\ S_{i}^{D_{65}} \end{array}\right],$$

where subscript *i* for cone excitations *L*, *M*, and *S* represents the color chip, instead of the cone classes in Equation (2), and superscript *j* represents the test illuminant. Subscript *k* for the coefficients (alpha, beta, and gamma) represents the lightness levels (3, 5, or 7) of the color chip. These coefficients were commonly used among the color chips of the same lightness *k* under illuminant *j*. The errors were defined based on the Euclidean distance between the estimated color and the matches of the subjects in the a^*^b^*^ plane of the CIE LAB space:

(4) $$\begin{array}{c} {\left[\Delta E_{i,j}^{*}\left(\alpha_{k}^{D_{65},j},\beta_{k}^{D_{65},j},\gamma_{k}^{D_{65},j}\right)\right]}^{2}\\ \;=[a_{i,j,new}^{*}\left(\alpha_{k}^{D_{65},j},\beta_{k}^{D_{65},j},\gamma_{k}^{D_{65},j}\right)\\ {\;-a_{i,j,match}^{*}\left(\alpha_{k}^{D_{65},j},\beta_{k}^{D_{65},j},\gamma_{k}^{D_{65},j}\right)]}^{2}\\ \;+[b_{i,j,new}^{*}\left(\alpha_{k}^{D_{65},j},\beta_{k}^{D_{65},j},\gamma_{k}^{D_{65},j}\right)\\ \;-{b_{i,j,match}^{*}\left(\alpha_{k}^{D_{65},j},\beta_{k}^{D_{65},j},\gamma_{k}^{D_{65},j}\right)]}^{2} \end{array}$$

where a^*^_i,j,new_ and b^*^_i,j,new_ represent the estimated chromaticity, derived from the estimated cone excitations $\hat{L}$^*j*^_*i*_, $\hat{M}$^*j*^_*i*_, and $\hat{S}$^*j*^_*i*_, as a function of the von-Kries coefficients (*alpha*, *beta*, and *gamma*) for color chips *i* under illuminant *j.* Likewise, a^*^_i,j,match_ and b^*^_i,j,match_ represent the matches of the subjects for color chips *i* under illuminant *j* in our previous study (Kuriki et al., 2000). Finally, three pairs of alpha, beta, and gamma values were obtained for the three lightness levels under illuminant *j* that minimizes the sum of the errors across the 20 color chips (Equation 5).

(5) $$\Delta E_{j}^{*}={\left(\sum_{i}\Delta E_{i,j}^{*2}\right)}^{1/2}$$

For comparison, similar errors were calculated between the estimated value and the actual matches from the results of our original study (Kuriki et al., 2000).

The increase or decrease of the coefficients by a common factor for all three photoreceptors (*alpha*, *beta*, and *gamma*) will result in changes in intensity, not chromaticity. To elucidate the effects of von-Kries-coefficient changes on the color appearance, the coefficients were normalized with respect to the beta value, i.e., the coefficient for the M-cone response, in the same way as in our previous study on the achromatic locus (Kuriki, 2006). The *beta*/*alpha* and *beta*/*gamma* values were used for changes to the achromatic point in the L-M and S directions of the cone-opponent space, respectively.

### Results

To determine the general trend through a comparison of our two studies (Kuriki et al., 2000; Kuriki, 2006), Figure 2 shows the results of the optimized coefficients for a subject who participated in both studies.

**Figure 2:**
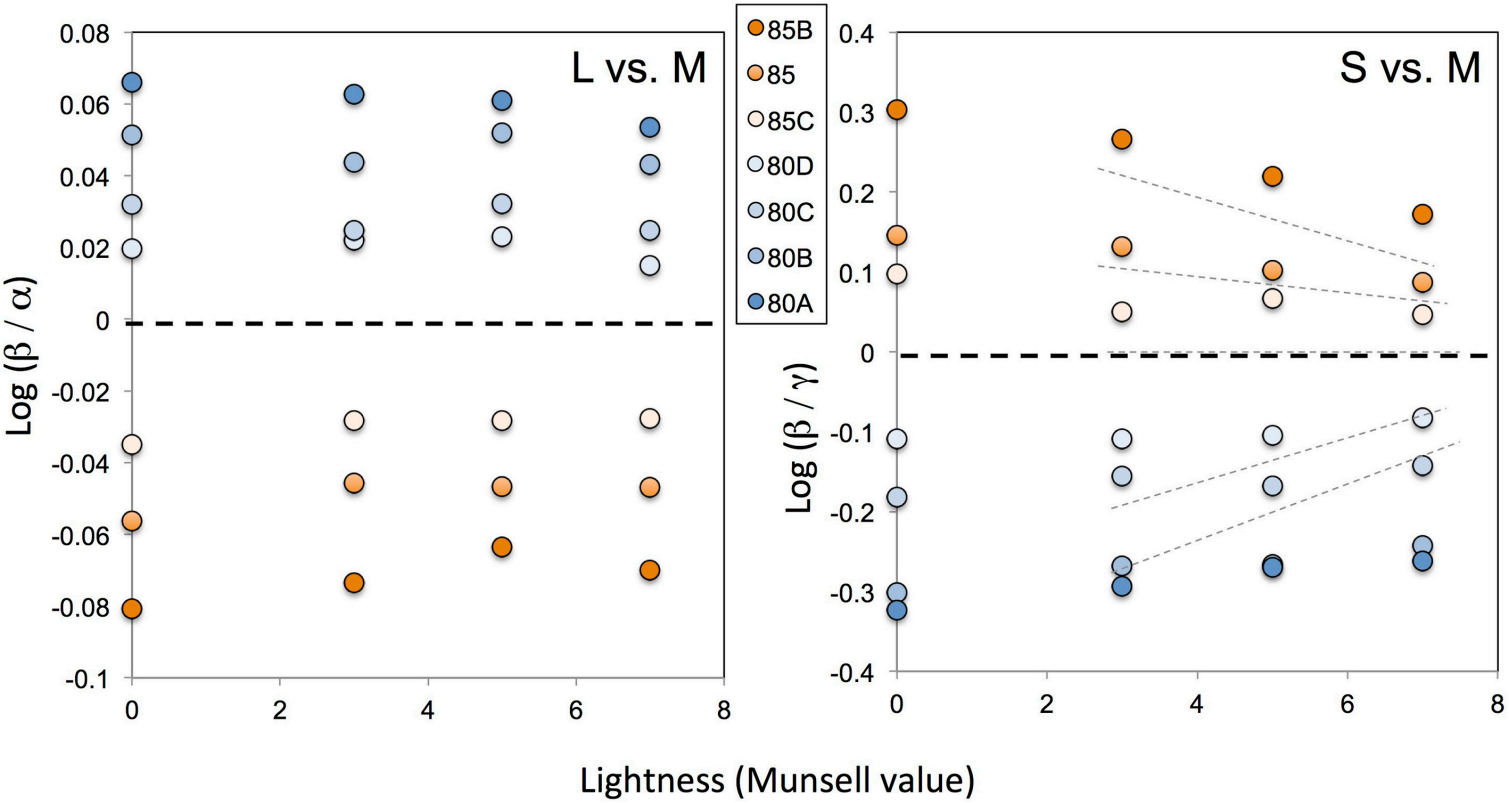
**Optimal von-Kries coefficients derived from our previous study (Kuriki et al., 2000), plotted in the space used for an evaluation of the relative cone responses (Kuriki, 2006)**. The horizontal axis indicates the lightness (in a Munsell value) of the color chips tested. The symbols on the vertical axis represent the chromaticity of the illuminant at this scale. The different symbols represent different illuminant conditions as indicated by the filter codes from Kodak (see Kuriki et al., 2000 for further details). Note that the panel on the left, comparing the relative contributions between the L- and M-cones, has a much smaller range than in our previous study (Kuriki, 2006) and between the S- and M-cones in the panel shown on the right. The thin dotted lines in the right panel show the achromatic-point loci re-plotted from our previous data (Kuriki, 2006) for the sake of comparison. Because the illuminant varied exclusively in the blue-amber direction, the relative contributions of the S- vs. M-cone responses show a similar trend as in the previous study.

Because the illuminant chromaticity in the color-matching study (Kuriki et al., 2000) changed in the blue-yellow direction, the changes were only evident in the results for the S-cone excitations. As a reference, the achromatic loci measured in our previous study (Kuriki, 2006) are shown by the dotted lines. The overall trend for the optimal von-Kries coefficient in our color-matching data (Kuriki et al., 2000) and the relative M-cone weights for the achromatic loci (Kuriki, 2006) are similar. This implies that the introduction of changes to the chromaticity of an achromatic point based on lightness may better explain the color shifts with regard to the von-Kries-like transformation, as reported in our achromatic-loci study (Kuriki, 2006).

**Figure 3:**
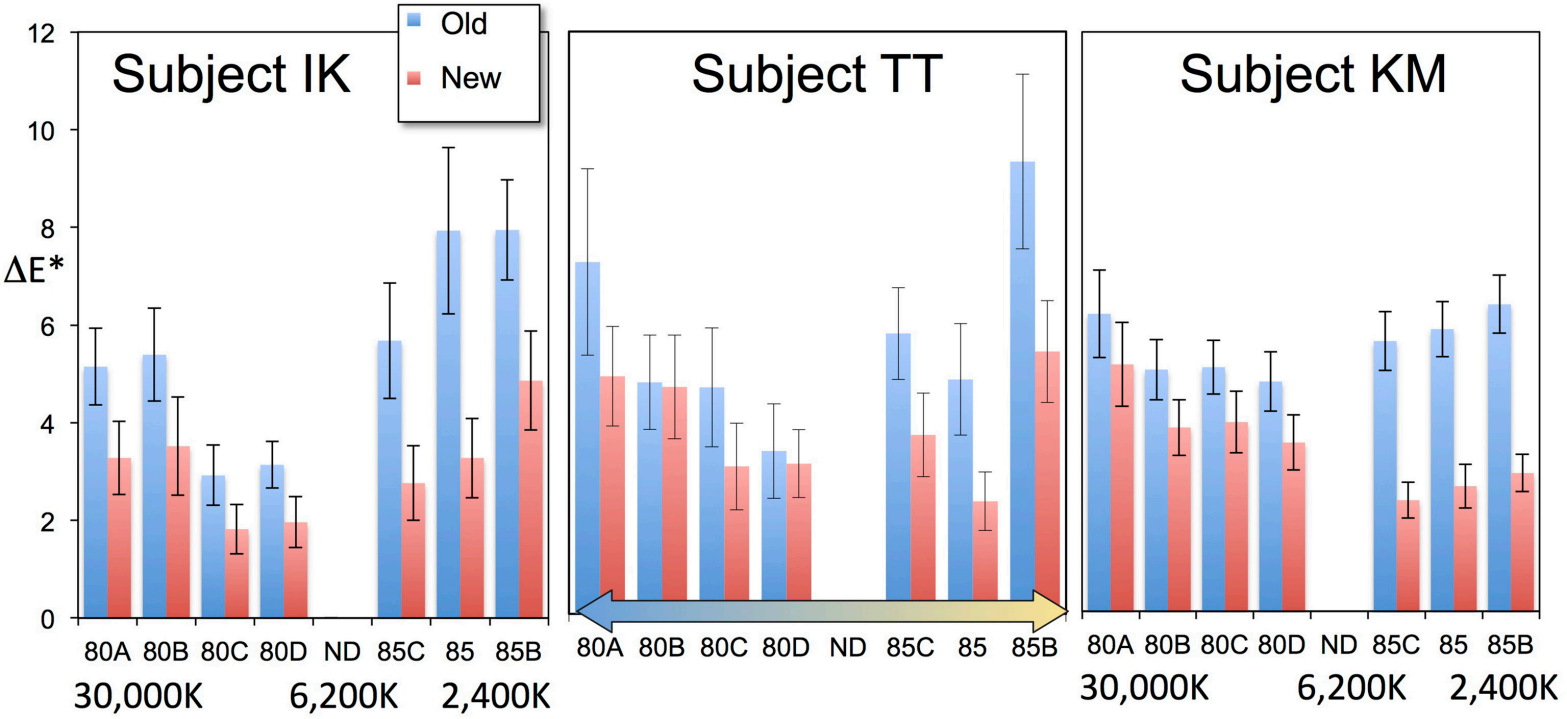
**Comparison of errors in our previous data (“Old”) against errors after optimizing for different lightness levels (“New”)**. Each bar represents the mean errors of 20 color chips, and the error bars represent the standard errors. The introduction of optimization for different lightness levels clearly improved the fitting. The two conditions show a statistically significant difference. See the text for further details.

To examine the efficiency of introducing achromatic point changes based on lightness, the residual errors in CIE delta-E^*^ were analyzed by comparing the von-Kries coefficient with and without the introduction of a lightness dependence. The results from the three subjects are shown in Figure 3. The absolute value of delta-E^*^ was reduced by approximately 40% on average. According to the analysis of variance, they differed with a statistical significance [*F*_(1, 829)_ = 88.5, *p* < 0.0001].

Our analysis described thus far has demonstrated that the lightness-level dependence of the relative changes in von-Kries coefficients (i.e., *relative M-cone weights*, *beta*/*alpha* and *beta*/*gamma*) better explains the results of asymmetric color matches measured using two different apparatuses for two entirely different purposes. In a recent study, another group tested and partially confirmed the dependence of achromatic points on the lightness of a target (Chauhan et al., 2014) using the relative M-cone weights defined in our previous study (Kuriki, 2006). Their results were slightly different from ours under one of their illuminant conditions, probably owing to the difference in the lightness level of the wall of the illuminated room, which acted as the largest adapting field for the subject, which was “dark” gray in Chauhan's et al. (2014) apparatus, and N5 in our own. These facts imply that a von-Kries transformation is an appropriate first-order approximation for color shifts under a different illuminant chromaticity, and improves significantly when the lightness dependence of the coefficients is introduced.

## Achromatic loci in color-appearance coordinates

### Purpose

As demonstrated in the previous section, progressive changes in the achromatic point with the lightness level were consistently observed in an asymmetric color-matching study conducted using a different apparatus, which was not designed to observe the achromatic-point shifts based on the lightness level. This implies that the shift in achromatic points with the lightness level may be a general trend. In a previous study, the achromatic loci were plotted in the axes based on the ratio of von-Kries coefficients among the cone types. This ratio (*relative M-cone weights* in Kuriki, 2006) was used to emphasize the lightness dependency of the von-Kries coefficients. If the von-Kries coefficients are independent from the lightness, the ratios of the cone excitations between different types will remain constant regardless of the lightness. Thus, the results shown in Figure 2 and those from our previous study (Kuriki, 2006) clearly demonstrate that they are lightness dependent.

As the next attempt, the achromatic loci were plotted in nonlinear spaces designed to represent the appearance of the color because they often introduce a lightness dependency in the formula (Fairchild, 2013). Among them, CIE LAB (1976) is a color coordinate with the simplest formula, which adopts the 1/3 power-law in terms of both lightness (L^*^) and chromaticity axes (a^*^, b^*^). If such a simple nonlinearity can account for the nonlinearity of an achromatic-point locus, it will appear in a simpler form in these color spaces.

### Methods

Data on the achromatic points from three subjects, measured in a previous study (Kuriki, 2006), were used in the plots. The achromatic loci were plotted in the following color spaces designed to take changes in the illuminant color into account: CIE LAB (1976), CIE CAM97s (revised version by Fairchild, 2001), CIE CAM02, Nayatani's model (Nayatani et al., 1990), Hunt's model (Hunt, 1994), and RLAB (Fairchild, 2013). These were used in two ways, i.e., based on their original method, and with normalization to daylight (D_65_).

The color appearance models use a standard “white point” to take illuminant-color changes into account. Basically, color appearance models use the tristimulus value of light reflected on a white (i.e., spectrally non-selective) surface with 100% reflectance under the environment illuminant of the test field as the normalization term. For example, the CIE XYZ (1931) tristimulus value of a spectrally non-selective (white) surface of 100% reflectance under the test illuminant is used as the normalization term [*X*_n_, *Y*_n_, *Z*_n_] for the derivation of the CIE LAB (1976) coordinates, which is an example of the original method mentioned above. The other method, normalization to daylight (D_65_), was realized by fixing the normalization term to the tristimulus value of 100% white under a D_65_ illuminant regardless of the actual illuminant conditions of the test field.

The achromatic-point loci from a previous study were plotted in various color spaces under both normalization methods to test whether achromatic loci in any of the color-appearance spaces show a much simpler locus representation.

### Results

**Figure 4:**
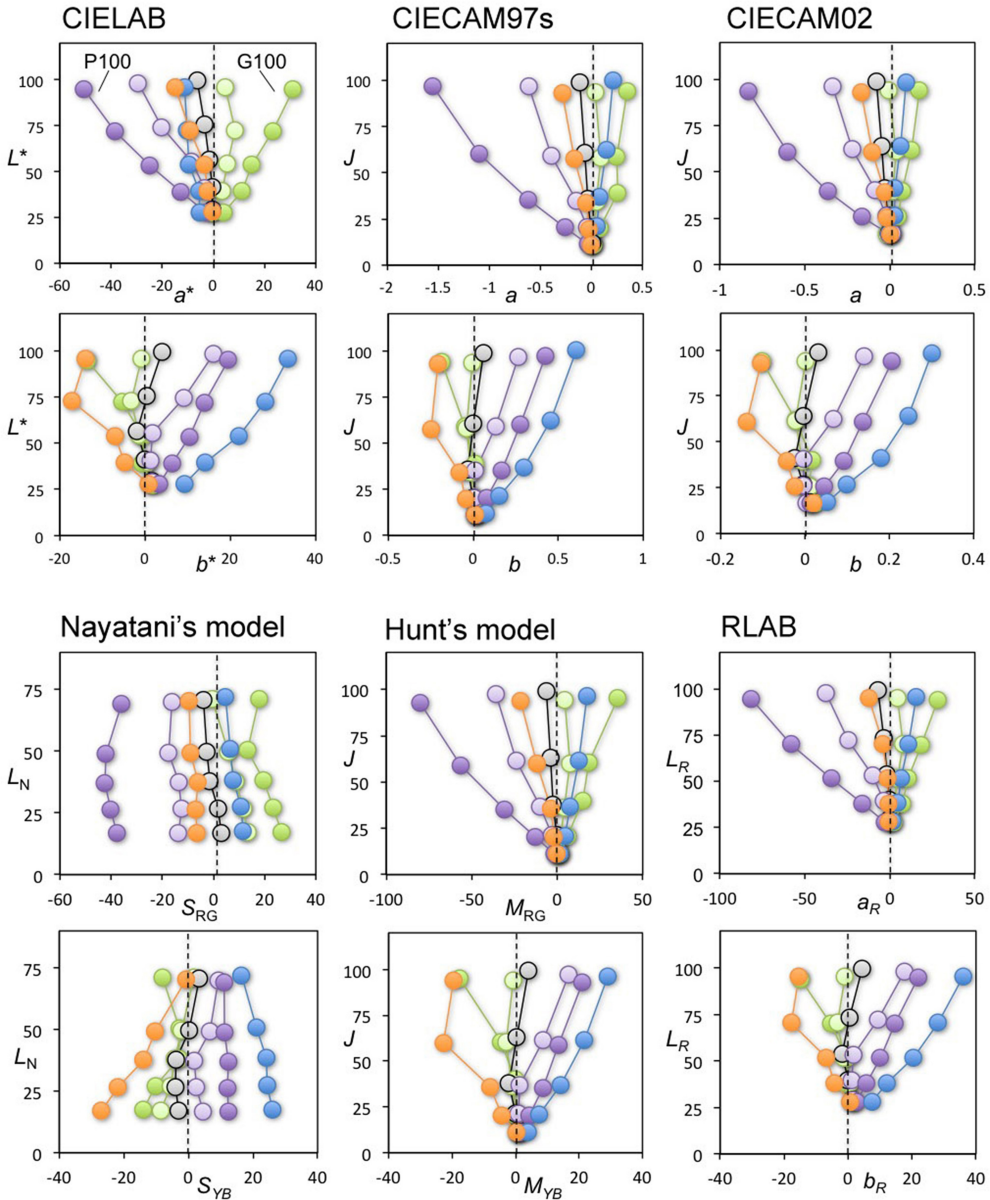
**Achromatic-point loci from our previous study (Kuriki, 2006) plotted in various spaces for color appearance**. Top-two rows, from the left: CIE LAB, CIE CAM97s revised version, and CIE CAM02. Bottom-two rows, from the left: Nayatani's model, Hunt's model, and RLAB. The symbols connected by a line represent the achromatic points under each illuminant condition, coded with a symbol color. For each color space, the horizontal and vertical axes in the panels in the upper row represent redness-greenness and lightness axes, respectively. Similarly, the horizontal and vertical axes in the panels in the lower row represent blueness-yellowness and lightness axes, respectively. With the exception of Nayatani's model, all plots show traces radiating from the zero point on the horizontal axis.

All panels in Figure 4 show the achromatic loci from one of the subjects in our previous study (Kuriki, 2006). A pair of two panels in a vertical arrangement show the results for each color space with lightness as the vertical axis, and red-green and yellow-blue as the horizontal axes in the upper and lower panels, respectively. The illuminant conditions for the achromatic loci measurements are indicated for the two most-saturated conditions (i.e., P100 and G100 in Kuriki, 2006) in the upper panel. They indicate that the achromatic loci for *reddish* and *greenish* illuminant conditions, counter intuitively, reside on the *negative* and *positive* sides of this plot, respectively. In most models, the loci spread out from the origin. Interestingly, the exception is Nayatani's model, which is the only color-appearance model known to implement changes to the color appearance based on lightness, i.e., the Helson-Judd effect. The loci in Nayatani's model space appear nearly parallel around the vertical axis. However, the loci do not coincide with the vertical axis in either the upper or lower panels, representing zero red-greenness and zero yellow-blueness, respectively. Rather, they tend to be located in a direction *opposite* from the illuminant color. For example, the achromatic loci for the purple illuminant are at the left-most part of the upper panel and on the right side in the lower panel, indicating that the achromatic surfaces (i.e., chromaticity along the vertical axis) should have appeared reddish and bluish. However, it should be repeated that the achromatic-point loci are the chromaticity of light that actually *appeared achromatic*. The purple illuminant clearly appeared reddish and bluish, even after 15 min of adapting to the illuminated room (see Kuriki, 2006 for details).

This result indicates that the illuminant color, which is almost at the horizontal position of the origin in this plot, appears as its illuminant color because the achromatic point indicates the chromaticity of light that appears colorless under the measurement conditions (Kuriki, 2006). The color spaces, however, were derived for an optimal description of the typical correspondence of color chips between illuminants A and D_65_ (Fairchild, 2013), and its origin does not guarantee a colorless appearance. This point is discussed further in Section General Discussions.

**Figure 5:**
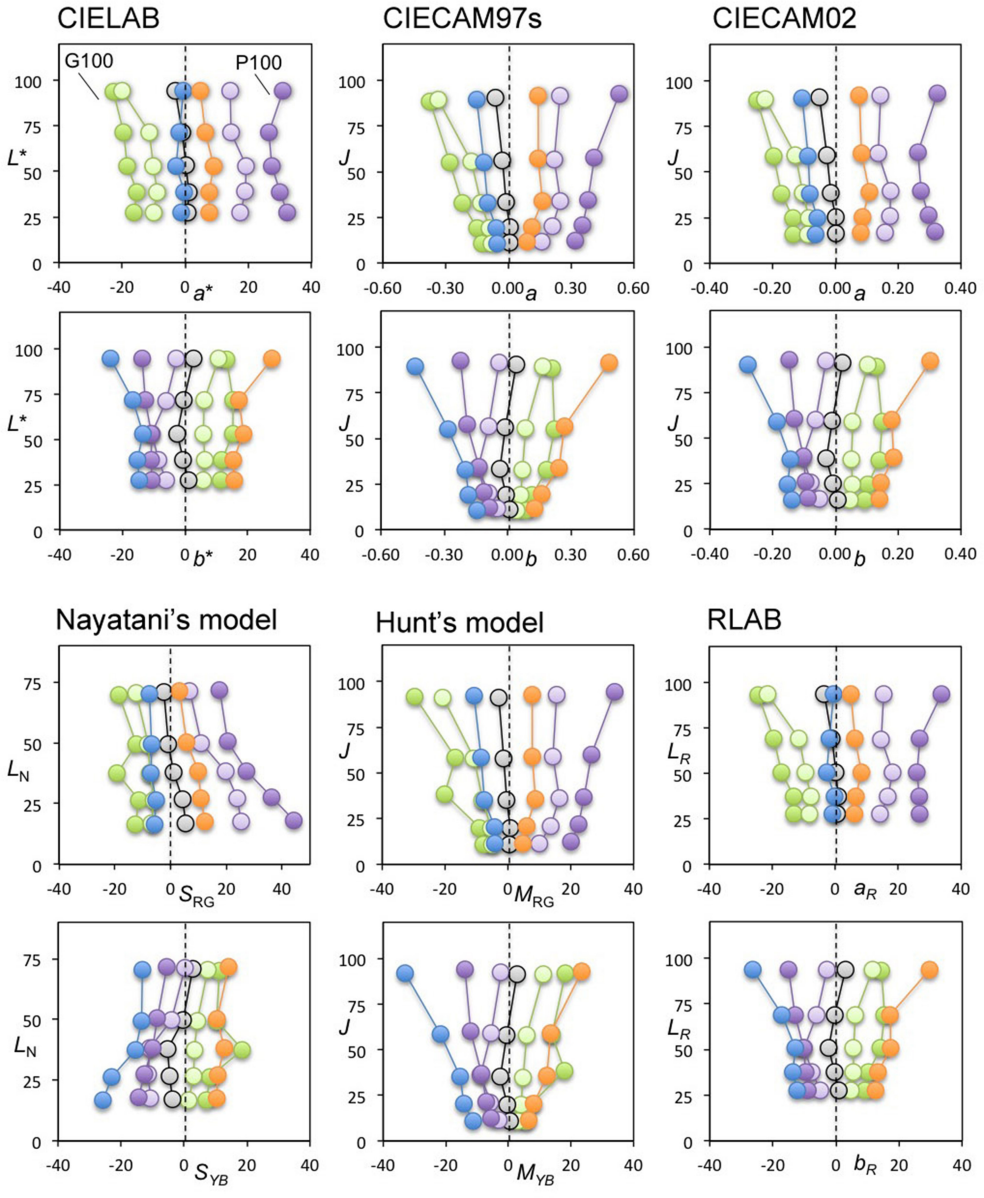
**The same achromatic-point loci shown in Figure 4 plotted in the color-appearance space, normalized to daylight (D_65_)**. The profiles show trajectories that are considerably more parallel than in Figure 4. See the text for further details.

Figure 5 shows the same loci in color spaces after normalization using a daylight (D_65_) illuminant without taking the illuminant conditions into consideration when plotting the achromatic loci. Interestingly, the loci in most of the color spaces resulted in a parallel arrangement similar to Nayatani's model shown in Figure 4. This result implies that the color appearance of the achromatic loci from the viewpoint of a color space normalized to D_65_ may be nearly constant in terms of chromatic saturation along with the lightness when the lightness dependence of the color appearance is taken into account.

To define the straightness of this plot (Figure 5), in comparison with a radial plot (Figure 4), the ratios of distance at the lightest and darkest points were calculated between the left- and right-most loci. Where the loci are parallel, and where for different illuminants they vary both systematically and in terms of smoothness, the distances along the horizontal axis at the lowest and highest lightness levels are nearly identical between the left- and right-most loci. Therefore, the most parallel plot will have the ratio closest to 1.0, as shown in the results listed in Table 1.

**Table 1:** **Parallelism of achromatic loci in various color spaces**.

	**CIELAB**	**CIECAM97s revised**	**CIECAM02**	**Nayatani**	**Hunt**	**RLAB**
Top/bottom: red-green	1.11	2.04	1.27	0.64	2.29	1.45
Top/bottom: yellow-blue	1.69	3.37	2.08	0.76	1.63	2.25
Average	1.40	2.70	1.68	0.70	1.63	2.25

On average, the color space with the ratio closest to 1.0 was CIE LAB. Nayatani's space reached nearly the same ratio, but its profile shrank slightly as the lightness increased (Table 1, Figure 5). The most important point illustrated in Figures 4, 5 is the general trend of the achromatic loci, which are nearly parallel in the color-appearance spaces normalized to daylight (D_65_), and in Nayatani's space after being normalized to the illuminant color. However, Nayatani's space normalized to the illuminant is the only exception among the color-appearance models, which are normalized to each ambient illuminant. Thus, it is natural to consider that the nearly straight locus of the achromatic points observed in the color-appearance space normalized to daylight may be a more general property.

We next plotted the achromatic loci for all three subjects from our previous study (Kuriki, 2006) in the CIE LAB space. CIE LAB space was selected as a representative color space among those used in Figures 4, 5 because it is the space with the simplest transformation and the simplest (i.e., parallel) phenomenal structure of achromatic loci, as shown in Figure 5. The CIE LAB coordinates used in Figure 5 were calculated through normalization using a diffuse white surface with 100% reflectance under a D_65_ illuminant as [*X*_n_, *Y*_n_, *Z*_n_]. Again, the achromatic-point loci for different illuminants were *not* normalized to a surface with 100% reflectance under each illuminant. Instead, the CIE LAB space normalized for D_65_ in Figure 5 was used for all illuminant conditions.

**Figure 6:**
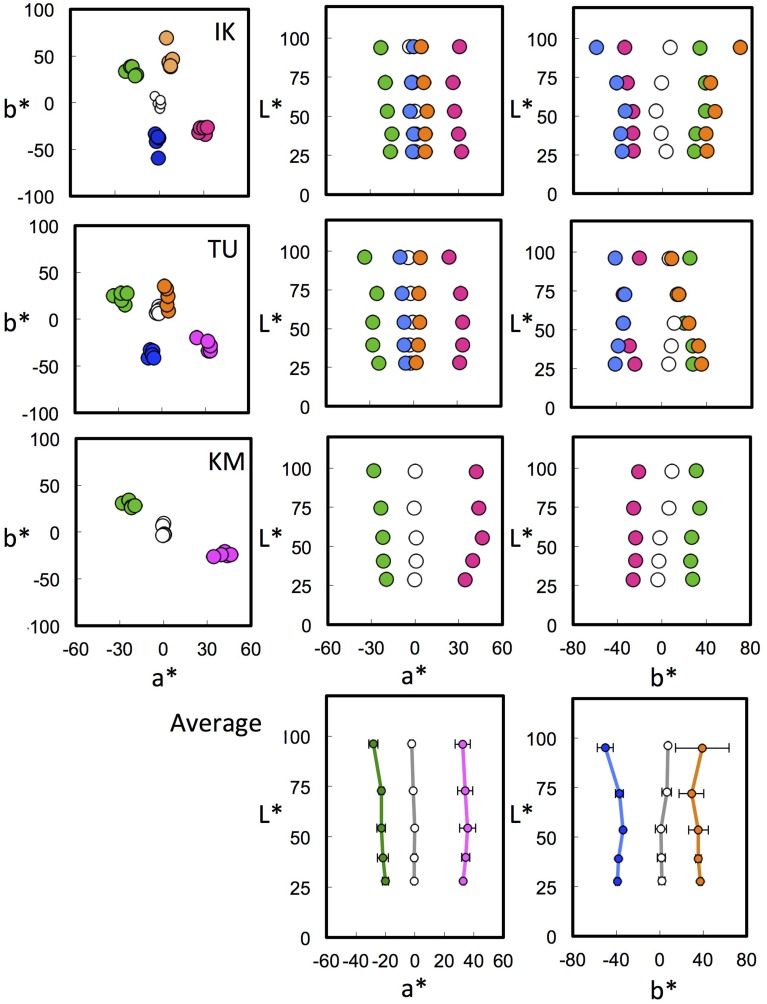
**Achromatic loci in CIE LAB space normalized to D_65_**. The top three rows show the chromatic loci for three subjects in the CIE LAB space normalized to daylight. The columns show different profiles, from left: a^*^-vs-b^*^, a^*^-vs-L^*^, and b^*^-vs-L^*^ planes. The various colors of the symbols represent a different illuminant chromaticity. For simplicity, the 50% green and 50% purple illuminant conditions (G50 and P50 in Kuriki, 2006) in IK were omitted. The bottom row shows the averaged achromatic loci in the a^*^-vs-L^*^ and b^*^-vs-L^*^ planes. The loci for different illuminant chromaticities are almost parallel to the vertical axis. See the text for further details.

Figure 6 shows the results of the achromatic-point loci from three subjects (Kuriki, 2006) plotted in a CIE LAB space normalized for D_65_. The loci of the averaged achromatic points are shown in the a^*^-vs-L^*^ or b^*^-vs-L^*^ plane. These planes reveal a clear parallelism between the achromatic-point loci and the L^*^ axis; the achromatic loci are nicely fitted as parallel shifts in the vertical lines. Furthermore, we informally tested the achromatic loci in the CIE LUV (1976) space, which also adopts the 1/3 power-law for lightness (L^*^) but not for chromaticity (u^*^, v^*^); however, a parallelism of the achromatic loci was not observed.

Nevertheless, the amount to which the point will shift away from the L^*^ axis is not easy to predict from the illuminant chromaticity, as shown in Figures 5, 6. The chromaticity loci for achromatic surfaces under each illuminant are radial lines from the origin in the a^*^-vs-L^*^ and b^*^-vs-L^*^ planes. A perceptual achromatic point, which appears non-chromatic, is clearly different from the chromaticity of the achromatic color chips except at around the lower L^*^ level.

A recent study reported that the iso-hue loci of four unique hues under chromatic illuminant conditions did not converge at a point, i.e., an achromatic point, and the estimated converging point was slightly offset from the lightness axis of CIE CAM02 (Xiao et al., 2014). One of the possible interpretations of the deviations of the achromatic point loci from the lightness axis, including our results in Figures 4, 5, are a failure in the prediction of an achromatic point by the color appearance models. However, the color appearance models are aimed to fit the apparent correspondence of color chips between different illuminants, by definition, and they do not use achromatic loci for the model optimization (Fairchild, 2013). Therefore, a discrepancy between the achromatic loci and the lightness axis of the color appearance models may be inevitable. In addition, it must be noted that the purpose of the present study is not to argue the “failure and/or success” of the color appearance models in terms of their prediction of the achromatic loci.

## General discussions

The first comparison between our two previous studies demonstrated that the dependence of the achromatic point on the lightness better explains the shifts in color appearance under different levels of illuminant chromaticity measured for entirely different purposes. This suggests that the color-appearance mechanisms may not be as simple as a cone-response scaling using a common coefficient for each cone class across all lightness levels. The fitting may be improved if the lightness dependency of the achromatic point (i.e., the scaling factor for each cone) is taken into account. This is because the lightness dependence of the achromatic-point loci is systematic, as discussed at length in our previous work (see Section 3.1 in Kuriki, 2006), with regard to comparisons with previous studies measuring unique yellows or achromatic points. However, a unified model that explains the achromatic loci under all illuminant conditions remains complicated (for examples of this, see Equations 7a and 7b and Figure 10 in Kuriki, 2006).

The second attempt demonstrated that the achromatic loci can be described in a relatively simple fashion (namely, a straight line) for color spaces that were designed to equate the perceptual differences based on the color appearance across a color space, such as CIE LAB (1976), which was defined to follow the 1/3 power-law for both lightness (L^*^) and chromaticity (a^*^ and b^*^). The achromatic-point loci were parallel to the L^*^ axis in the CIE LAB space when normalized to daylight. A similar trend was observed in other spaces designed to predict color shifts under different levels of illuminant chromaticity when normalized to daylight. Nayatani's model was the only exception, showing somewhat parallel lines in a space normalized under each illuminant chromaticity. However, the other spaces also show nearly parallel loci for the achromatic points when normalized to daylight. Thus, it is natural to interpret the parallelism under daylight normalization being a more general characteristic.

What does it mean if a line parallel to a lightness axis *normalized to daylight* can best represent an achromatic locus? A locus in a color-appearance space parallel to the lightness axis represents an iso-chromatic line, i.e., a line of coordinates with *constant hue and saturation components across the lightness*. The color space normalized to daylight implies that the achromatic locus is defined in a color space for which the chromaticity of the illuminant is irrelevant. However, the direction and offset from the lightness axis are defined based on the environmental illuminant. This implies that a system invariant to changes in the environmental illuminant defines the color space normalized to daylight, but not the color appearance.

This implication applies when the visual system holds a color axis invariant to the environmental illuminant. Such a high degree of color constancy was observed when tested using a real object and categorical judgment (Uchikawa et al., 1998; Olkkonen et al., 2009). In general, however, color-matching results exhibited a color constancy that remained incomplete (see Foster, 2011 for a systematic review along with the original derivation of the constancy index for each study). Further, this can be confirmed from the observation of a sheet of white paper being identifiable under candlelight despite the fact that it does not appear as white as it would in daylight. From a computational point of view, a nearly color-constant property is present in the scene, i.e., the contrast of the cone responses among the surfaces is nearly invariant across changes in illuminant color (e.g., Land and McCann, 1971; Lucassen and Walraven, 1993). A color-appearance space normalized to daylight may be defined for the human visual system if such information is fully utilized.

**Figure 7:**
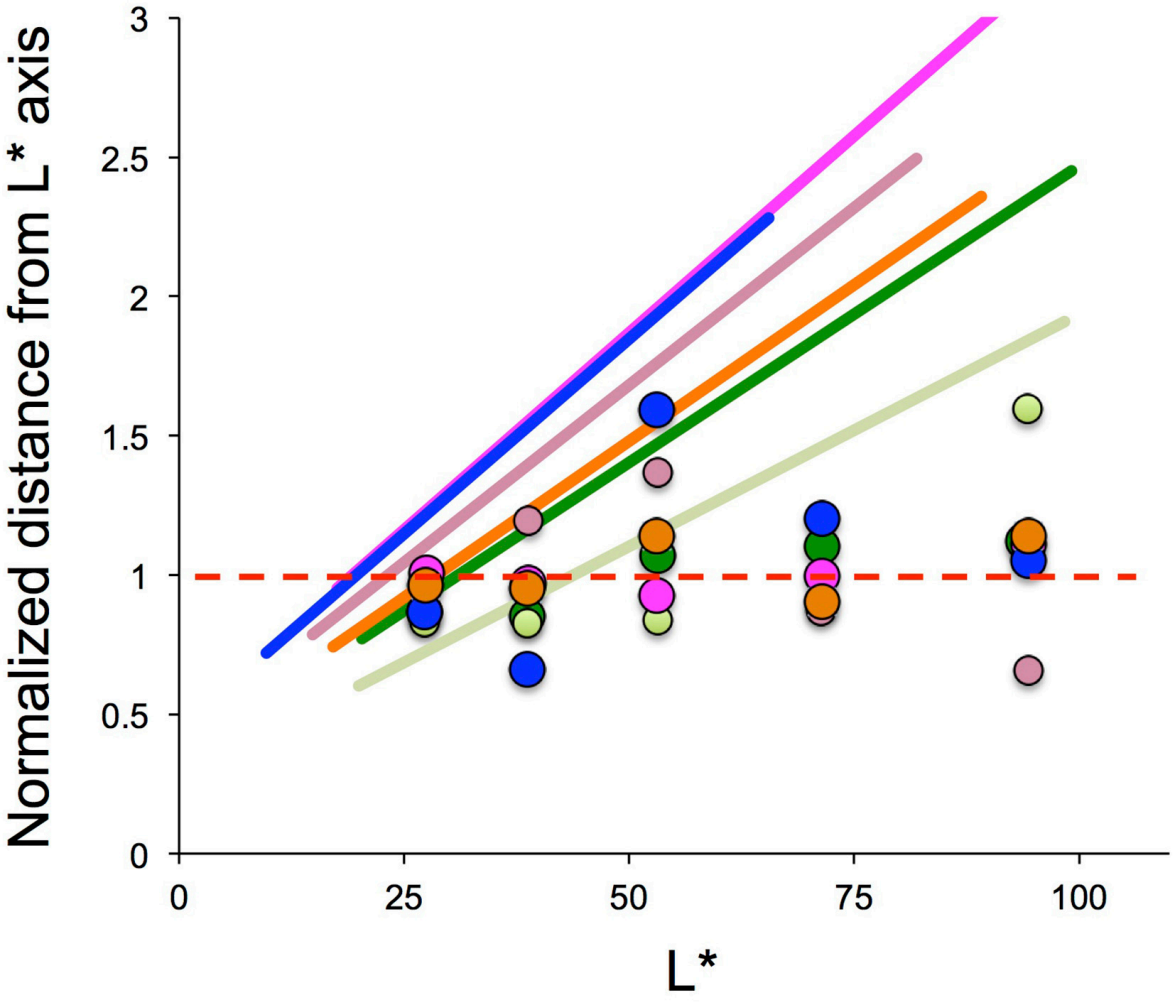
Illuminant chromaticity profile as a function of lightness (L^*^). The vertical axis shows the Euclidean distance of the illuminant chromaticity from the lightness axis (a^*^, b^*^) = (0,0) for each a^*^-vs-b^*^ plane normalized to the Euclidean distance for the achromatic points averaged across five lightness levels. The slanted lines indicate the illuminant chromaticity, and the intersection at 1.0 is found at approximately L^*^ = 25. See the text for further details.

Given that a color-appearance space normalized to daylight was obtained, an open question remains regarding how to determine the position of the achromatic locus. A clue to answering this question may be found in the achromatic point under a *low level of lightness*. Whereas, many studies have reported that color-appearance mechanisms are incomplete with regard to color constancy, there are no clear answers to how this incompleteness is determined. A clue to this can be found in the results of the present study under a low level of lightness. Figure 7 provides a profile of the illuminant chromaticity in relation to the achromatic point-locus in a CIE LAB space. The loci of the achromatic point and the illuminant chromaticity are projected onto a plane that includes the L^*^ axis and the illuminant-chromaticity locus. The deviation along the vertical axis indicates the chromaticity normalized to the achromatic points averaged across the various lightness levels under each illuminant condition.

These lines intersect at a value of 1.0 on the vertical axis around the darkest level of lightness. If the color-appearance space, normalized to daylight, is acquired before judging the color appearance, the neutral point in the hue-saturation space can be defined by the illuminant chromaticity at the lower end of the lightness. The slanted lines for the illuminant chromaticity have a value of 1.0 or less only with a very dark range of lightness. Because a value of 1.0 in the vertical axis represents an achromatic point, and a larger value represents greater saturation in the illuminant hue, an achromatic sample that reflects the same illuminant chromaticity with a value of less than 1.0, as shown in Figure 7, would appear in the opposite hue of the illuminant. The original work by Helson (1938) reported the appearance of the opposite hue for darker samples observed within a much wider range of lightness. However, subsequent work by Helson and Michels (1948) reported that the achromatic point for the darkest level of lightness appeared to be nearly the same as the illuminant color. This was described in terms of *adaptation ratio r* (Figure 2 in their work), and is qualitatively similar to the results from our previous study (Figure 10 in Kuriki, 2006). The adaptation ratio *r* rarely took a value higher than 100%, implying that the samples appearing in the opposite hue were rare in the study by Helson and Michels (1948). Therefore, further studies are required to investigate the means by which the achromatic points are to be determined.

One clear weak point of the present study is the small number of subjects used, i.e., three. It is also true that there are some minor deviations from the ideal parallelism, which is different among the subjects. However, the general trend after normalization is similar across the subjects and illuminant chromaticity (Figures 6, 7). In addition, despite the minor differences, the general tendency of the “approximately parallel form” of achromatic loci in the CIELAB space, normalized to daylight, is mostly consistent across the subjects under most illuminant conditions, i.e., 15 loci in total from the three subjects. Because the aim of the latter half of this manuscript is to share this phenomenon and the conceptual idea regarding the cause of this achromatic loci, the results of the present study, although from a small number of subjects, can be considered a useful hint toward areas of future study on achromatic point loci.

### Conflict of interest statement

The author declares that the research was conducted in the absence of any commercial or financial relationships that could be construed as a potential conflict of interest.
